# Evidence of potentially unrelated AmpC beta-lactamase producing *Enterobacteriaceae* from cattle, cattle products and hospital environments commonly harboring the *bla*_ACC_ resistance determinant

**DOI:** 10.1371/journal.pone.0253647

**Published:** 2021-07-29

**Authors:** Keduetswe Matloko, Justine Fri, Tshepiso Pleasure Ateba, Lesego G. Molale-Tom, Collins Njie Ateba

**Affiliations:** 1 Antimicrobial Resistance and Phage Biocontrol Research Group, Department of Microbiology, School of Biological Sciences, Faculty of Natural and Agricultural Sciences, North-West University, Mmabatho, South Africa; 2 Food Security and Safety Niche Area, Faculty of Natural and Agricultural Sciences, North-West University, Mmabatho, South Africa; 3 Centre for Animal Health Studies, Faculty of Natural and Agricultural Sciences, North-West University, Mmabatho, South Africa; 4 Unit for Environmental Sciences and Management - Microbiology, North-West University, Potchefstroom, South Africa; Suez Canal University, EGYPT

## Abstract

The occurrence and genetic relatedness of AmpC beta-lactamase producing *Enterobacteriaceae* isolated from clinical environments, groundwater, beef, human and cattle faeces were investigated. One hundred seventy-seven (177) samples were collected and cultured on MacConkey agar. A total of 203 non-repetitive isolates were characterised using genus/species-specific PCRs and the identified isolates were subjected to antibiotic susceptibility testing. The production of AmpC beta-lactamases was evaluated using cefoxitin disc, confirmed by the D96C detection test and their encoding genes detected by PCR. The D64C extended-spectrum beta-lactamases (ESBL) test was also performed to appraise ESBLs/AmpC co-production. The genetic fingerprints of AmpC beta-lactamase producers were determined by ERIC-PCR. A total of 116 isolates were identified as *E*. *coli* (*n* = 65), *Shigella* spp. (*n* = 36) and *Klebsiella pneumoniae* (*n* = 15). Ciprofloxacin resistance (44.4–55.4%) was the most frequent and resistance against the Cephem antibiotics ranged from 15–43.1% for *E*. *coli*, 25–36.1% for *Shigella* spp., and 20–40% for *K*. *pneumoniae*. On the other hand, these bacteria strains were most sensitive to Amikacin (0%), Meropenem (2.8%) and Piperacillin-Tazobactam (6.7%) respectively. Nineteen (16.4%) isolates comprising 16 *E*. *coli* and 3 *Shigella* spp. were confirmed as AmpC beta-lactamase producers. However, only *E*. *coli* isolates possessed the corresponding resistance determinants: *bla*_ACC_ (73.7%, *n* = 14), *bla*_CIT_ (26%, *n* = 5), *bla*_DHA_ (11%, *n* = 2) and *bla*_FOX_ (16%, *n* = 3). Thirty-four (27.3%) *Enterobacteriaceae* strains were confirmed as ESBL producers and a large proportion (79.4%, *n* = 27) harboured the *bla*_TEM_ gene, however, only two were ESBLs/AmpC co-producers. Genetic fingerprinting of the AmpC beta-lactamase-producing *E*. *coli* isolates revealed low similarity between isolates. In conclusion, the findings indicate the presence of AmpC beta-lactamase-producing *Enterobacteriaceae* from cattle, beef products and hospital environments that commonly harbour the associated resistance determinants especially the *bla*_ACC_ gene, nonetheless, there is limited possible cross-contamination between these environments.

## 1. Introduction

The family *Enterobacteriaceae* consists of bacterial species frequently isolated from clinical specimens and most often incriminated in a variety of infections [1, 2]. The natural hosts of these organisms are ruminants and therefore, may be found in food, water and the environment from where transmission to humans may occur especially when proper hygienic procedures are compromised. Most *Enterbacteriaceae* were known to be non-pathogenic, however, over the years some members including strains/serotypes of *E*. *coli*, *Shigella* spp. and *Klebsiella pneumoniae* have become virulent accounting for diarrheal cases (which is a major burden in low and middle-income countries), other intra-abdominal, urinary tract, and bloodstream infections, as well as hospital and healthcare-associated pneumonia [3]. Pathogenic *E*. *coli* strains classified based on their virulence properties including enterotoxigenic *E*. *coli*, ETEC; enterohemorrhagic *E*. *coli*, EHEC; enteroinvasive *E*. *coli*, EIEC; enteropathogenic *E*. *coli*, EPEC; enteroaggregative *E*. *coli*; EAEC and diffusely adherent *E*. *coli* (DAEC), neonatal meningitis *E*. *coli* (NMEC), uropathogenic *E*. *coli* (UPEC), and avian pathogenic *E*. *coli* (APEC) cause mild to severe food poisoning in their hosts as well as may lead to extra-intestinal illnesses [4, 5]. A serotype such as the Shiga toxin-producing *E*. *coli* 0157:H7 has been associated with life-threatening diseases such as haemolytic uremic syndrome (HUS), haemorrhagic colitis, and thrombotic thrombocytopenic purpura which may be fatal. Four *Shigella* spp., S. *flexneri*; *S*. *dysenteriae*; *S*. *boydii* and *S*. *sonnie* have been described and are thought to have evolved from ancestral non-pathogenic *E*. *coli* by the acquisition of a large virulence plasmid [6], that encodes many virulence factors on its *ipa-mxi-spa* region of which the invasion plasmid antigen (antigens (IpaA, IpaB, IpaC and IpaD proteins) is one of the most essential. These proteins are important for bacterial invasion of epithelial cells as well as immune cell escape [7]. Infection caused by *Shigella* spp., Shigellosis is more common among children and travellers and approximately 60% of cases are accounted for by S. *flexneri*, although infection with *S*. *dysenteriae* causes greater toxicity. *S*. *dysentariae* serotype 1 produces the Shiga toxin which can lead to HUS and eventually cause hemolytic anaemia, uremia and thrombocytopenia with up to 20% mortality. *Shigella* infection can also activate reactive arthritis [8]. *K*. *pneumoniae* on the other hand occurs as an opportunistic pathogen especially in persons with weakened immune systems and cause soft tissue (e.g. cellulitis and necrotizing fasciitis), to life-threatening infections such as pneumonia, septicemia, meningitis, and endophthalmitis as well as are implicated in a significant number of cases of hospital-acquired urinary tract infections [9].

There is an increased report of multidrug resistance globally that is considered a public health threat. Several previous studies revealed the emergence of multidrug-resistant bacterial pathogens from different origins including animals, fish, birds, drinking water and other environmental water sources that may be sources of transmission through the food chain to human consumers resulting in severe illness [10–17]. *Enterobacteriaceae* have exhibited varied antibiotic resistance mechanisms such as the intrinsic production of beta-lactamases that inactivates beta-lactam antibiotics in predominance thus proving to be a public health threat [18]. Of these, the production of Extended Spectrum Beta Lactamases (ESBL) and AmpC beta-lactamases have been the most prominent [19]. AmpC beta-lactamases are group 1 or class C cephalosporinases mostly encoded on chromosomal sequences of many *Enterobacteriaceae* and mediate resistance to cephalothin, cefazolin, cefoxitin and most penicillins, as well as enhance the production of β-lactamase inhibitor-β-lactam combinations [20]. Chromosomally encoded AmpC genes are constitutive and typically expressed at low levels, however, in most *Enterobacteriaceae* these genes are inducible and overexpressed in response to β-lactam or other stimuli conferring resistance to broad-spectrum Cephalosporins including Ceftriaxone, Ceftazidime, and Cefotaxime [21, 22]. *Enterobacteriaceae* belonging to the CESP group or group II *Enterobacteriaceae* (*Citrobacter*, *Enterobacter*, *Serratia* and *Providencia*) alongside *Hafnia* and *Morganella* included more recently, are typical examples with chromosomally-encoded AmpC beta-lactamases [11, 22]. However, in other *Enterobacteriaceae*, such as *E*. *coli* and *Shigella* spp., AmpC β-lactamases are chromosomally encoded and constitutively low or poorly expressed but non-inducible. In these bacterial species, expression of AmpC beta-lactamases is due to hyperproduction as a result of chromosomal *ampC* gene mutation or expression of plasmid-encoded AmpCs [21]. There is an increasing interest in transmissible (plasmid)-mediated cephalosporinases due to their mobility and potential spread. Species harbouring *ampC* positive plasmids confer resistance to beta-lactams comparable to their chromosomal equivalents and include *E*. *coli*, *Salmonella* spp., *Klebsiella pneumoniae*, *Citrobacter freundii*, *Proteus mirabilis*, and *Enterobacter aerogenes* [23, 24]. Currently, resistance amongst these members of *Enterobacteriaceae*, particularly *E*. *coli* to cephalosporins, beta-lactamase inhibitors and cephamycins is concerning and presents a huge challenge to public health.

Although AmpC β-lactamases usually hydrolyse a wide variety of β-lactam antibiotics except for the carbapenems and cefepime, unfortunately, overproduction of AmpC coupled with the outer membrane porin mutations can reduce susceptibility to carbapenems, particularly in plasmid-mediated AmpC producers [22]. This is a cause for concern, as carbapenems are first-line drugs in cases of antibiotic resistance [25], leaving limited available therapeutic options against MDR pathogens. It is therefore imperative to determine the frequency of occurrence of AmpC beta-lactamase-producing bacteria and the possible transmission of their resistance traits between environments.

Various phenotypic tests including the AmpC disk test, modified three-dimension test (M3DT), boric acid detection test, double disc synergy test (CC-DDS) and the D69C AmpC detection set have been reliably used in the detection of AmpC-producing traits in bacterial strains [26–30]. These tests have been supplemented with genotypic tests especially for the detection of plasmid-mediated AmpC genes which pose a significant threat to public health. Genotypic tests have been described for six *ampC* gene families (MOX, CIT, EBC, FOX, DHA and ACC-1) for which discriminative phenotypic tests are yet to be developed [31]. Despite the reported evidence on the increasing detection of AmpC beta-lactamases among *Enterobacteriaceae* worldwide [22, 32, 33], little or no data has been generated for South African strains and this highlights the importance of the present study. In the current study area, strains belonging to the family *Enterobacteriaceae* have previously been isolated from various sources and their antimicrobial susceptibility profiles determined [34–38]. Moreover, previous studies have indicated that besides humans and livestock, food products can also harbour AmpC beta-lactamases-producing Gram-negative bacteria [2, 22, 30, 39–42]. However, besides the study undertaken by Coertze and Bezuidenhout [43], there is no documented data of AmpC beta-lactamases-producing *Enterobacteriaceae* in the North-West Province of South Africa. This also raises the need to screen community-derived samples for the resistance traits in the area. The study was therefore aimed to isolate, identify and characterise AmpC beta-lactamase-producing *Enterobacteriaceae* isolated from cattle/beef products, hospital environments, groundwater and humans and to determine their genetic relatedness.

## 2. Materials and methods

### 2.1 Sampling

A variety of samples totalling 177 were randomly collected in Kareefontein, Lichtenburg, Mafikeng, Rustenburg and Zeerust in the North-West Province, of South Africa. Ethical approval for the study was obtained from the North-West University Health Science Ethics Committee (NWU-00728-18-A9) and permission obtained from the North West Provincial Health Department, South Africa. A verbal informed consent was obtained from parents/guardians of minors, from which feacal samples were collected, however documented in the laboratory note book. Fifty-four (54) faecal samples were collected directly from the rectum of cattle using sterile arm-length gloves. Twelve (12) beef samples that comprised wors, mincemeat, beef polony and beef fillet steaks were randomly collected from different butcheries and retail stores. Fifty-four (54) water samples were collected from boreholes in villages following the recommendations for groundwater sampling [44]. Seven (7) human stool samples were collected from patients aged between 4 months and 4 years at the Mafikeng Provincial hospital, North West Province of South Africa. In addition, sixty-four (64) swab samples were collected from clinics around the North-West Province of South Africa. Swab samples were obtained from consultation rooms, reception area, staff kitchen and patient toilets at noon after personnel and patients had been in contact with the surfaces. All samples were collected into appropriate containers and transport media where possible and transported on ice to the laboratory for analysis within six hours of collection. Table 1 shows the numbers of the samples collected from various sources.

**Table 1:** Types and numbers of the samples collected during the study.

Sample type	Sampling area	No. of samples	Total
Groundwater	Mafikeng	24	40
	Rustenburg	8	
	Zeerust	7	
	Kareefontein	1	
Beef	Lichtenburg	6	12
	Zeerust	5	
	Kareespruit	1	
Hospital surface swabs	Bophelong	9	64
	Danville	10	
	Lonely park	7	
	Motlhabeng	18	
	Ramatlabama 600	8	
	Unit 9	12	
Cattle faeces	Zeerust	54	54
Human faeces	Mafikeng	7	7

### 2.2 Isolation and identification of members of *Enterobacteriaceae*

#### 2.2.1 Sample processing

Beef products, human and cattle faeces were processed as previously described [45, 46]. Briefly, 25 g of cattle and human faeces were homogenized in 225 ml of 0.1% (w/v) buffered peptone water (BPW) while 1 g of beef sample was washed in 10 ml of 2.0% (w/v) BPW (Lab M Limited, UK). The cotton wool swabs were washed in 10 ml of 2% (w/v) peptone water and vortexed. For groundwater samples, aliquots of 100 ml of each sample were aseptically filtered through 0.45 μm pore-size filters (Whatman Laboratory Division, Maidstone, England) as recommended [47].

#### 2.2.2 Isolation of members of *Enterbacteriaceae*

One hundred microliters of each suspension was spread-plated on MacConkey agar with crystal violet (Biolab, SA) for the isolation of *Enterbacteriaceae* [48]. Membrane filters were also aseptically inoculated on the agar. Plates were incubated aerobically at 37 °C for 24 hours. One to three colonies per positive plate depending on differing but typical morphological characteristics of *Enterobacteriaceae* were sub-cultured on fresh agar for purity. A 25% glycerol stock was prepared from a fresh overnight pure culture in tryptic soy broth and preserved at −80°C for future analysis.

#### 2.2.3 Molecular identification of members of *Enterbacteriaceae*

Bacterial chromosomal DNA was extracted from presumptive *Enterobacteriaceae* isolates using the CTAB method [49]. As an internal control, bacterial 16S rRNA gene of all presumptive isolates was amplified by PCR. using the universal oligonucleotide primer sequences 27F 5’-AGA GTT TGA TCM TGG CTC AG-3’ and 1492R 5’-TAC GGY TAC CTT GTT ACG ACT T-3’ Polymerase reaction assays were also used for genus/species-specific identification of *Enterobacteriaceae* of interest: *E*. *coli*, *Klebsiella pneumoniae*, and *Shigella* spp. (Table 2 consist of all oligonucleotide primers used in the study). PCR assays were performed in a C1000 Touch Thermal Cycler (Bio-Rad, California, USA) and for each reaction, a total volume of 25 μl containing 12.5 μl DreamTaq Master Mix (1X PCR buffer, 2 mM MgCl_2_, 0.6 units of *Taq* DNA polymerase and 0.2 mM of each dNTPs), 11 μl of nuclease-free water, 0.5 μl oligonucleotide primer set (0.1μM final reaction concentration) and 1 μl of DNA template was prepared. *E*. *coli* ATCC 35218 and other in-house strains previously confirmed positive for the other targeted strains were used as controls. Otherwise mentioned, all DNA and amplicons resulting from PCR assays in this study were resolved by electrophoresis on a 1% (w/v) agarose gel stained with 0.001mg/ml ethidium bromide (BioRad, UK). Gel electrophoresis was carried out at 70V and 250A for 90 minutes in 1 X TAE buffer (40 mM Tris, 1 mM EDTA and 40 mM glacial acetic acid, pH 8.0). Gels were visualized under UV light at a wavelength of 420 nm and the images were captured using a ChemiDoc Imaging System (Bio-Rad ChemiDoc^™^ MP Imaging System, California, USA).

**Table 2:** Oligonucleotide primer sequences used in the study.

Gene Target	Primer sequences (5’-3’)	Product size (bp)	Annealing temperature	Reference
*16S rRNA*	27F: AGAGTTTGATCMTGGCTCAG	1420	55 °C	[52]
	1492R: TACGGYTACCTTGTTACGACTT			
*E*. *coli uidA* gene	UAL: TGGTAATTACCGACGAAAACGGC	147	50°C	[53]
	UAR: ACGCGTGGTTACAGTCTTGCG			
*K*. *pneumoniae* 16S-23S spacer	PF: ATTTGAAGAGGTTGCAAACGAT	130	55 °C	[54]
	PA: TTCACTCTGAAGTTTTCTTGTGTTC			
*Shigella* spp. putative integrase gene	GF: TCCGTCATGCTGGATGAACGATGT	159	60 °C	[55]
	GR: ACAGTTCAGGATTGCCCGAGACACA			
*Bla*_TEM_	F: AAACGCTGGTGAAAGTA	822	45°C	[56]
	R: AGCGATCTGTCTAT			
***AmpC* genes**				
MOX-1, MOX-2, CMY-1 CMY-8 to CMY-11	MOXMF: GCTGCTCAAGGAGCACAGGATGAT	520	55.9 °C	[31]
	MOXMR: CACATTGACATAGGTGTGGTGC			
LAT-1 to LAT-4, CMY-2 to CMY-7, BIL-1	CITMF: TGGCCAGAACTGACAGGCAAA	576		
	CITMR: TTTCTCCTGAACGTGGCTGGC			
ACC	ACCMF: AACAGCCTCAGCAGCCGGTTA	403		
	ACCMR: TTCGCCGCAATCATCCCTAGC			
MIR-1T ACT-1	EBCMF: TCGGTAAAGCCGATGTTGCGG	302		
	EBCMR: CTT CCACTGCGGCTGCCAGTT			
FOX-1 to FOX-5b	FOXMF: AACATGGGTATCAGGGAGATG	190		
	FOXMR: CAAAGCGCGTAACCGGATTGG			
DHA-1, DHA-2	DHAMF: AACTTTCACAGGTGTGCTGGGT	619		
	DHAMR: CCGTAC GCATACTGGCTTTGC			

### 2.3 Antimicrobial susceptibility testing

The antimicrobial resistance profiles of the isolates were determined by the disc diffusion assay on Mueller Hinton agar following the CLSI (Clinical Laboratory Standards Institute) recommendations [50]. Twelve antibiotics (Mast Diagnostics, UK) belonging to seven groups were used: β-lactam/ β-lactamase inhibitor (Piperacillin-tazobactam, 100 μg /10 μg), Carbapenem (Meropenem, 10 μg), Monobactam (Aztreonam, 30 μg), Aminoglycoside (Gentamicin, 10 μg; Amikacin, 30 μg), Quinolone (Ciprofloxacin), Cephem (Cefepime, 30 μg; Cefotaxime, 30 μg; Ceftriaxone, 30 μg; Cefuroxime, 30 μg and Ceftazidime, 30 μg) and folate pathway inhibitor (Trimethoprim-Sulfamethoxazole, 1.25/ 23.75 μg). These antimicrobial agents were selected based on resistance data from some previous studies conducted in the study area [15, 50]. In addition, third-generation cephalosporins were included in the evaluation due to the fact that they have a wide activity against gram-negative microorganisms and are very useful for the treatment of serious bacterial infections in humans. The resulting growth inhibition zone diameters were used to classify isolates as resistant, intermediate resistant and susceptible based on the CLSI breakpoint values [51]. Isolates presenting intermediate resistant patterns were considered as potential resistant isolates.

### 2.4 Phenotypic and genotypic detection of AmpC β-lactamases and ESBL

Phenotypic detection of AmpC β-lactamase production was carried out using cefoxitin disc (30 μg) as previously described [22, 30]. *E*. *coli* (ATCC 35218), a beta-lactamase-producing strain was used as a positive control. Isolates exhibiting resistance to cefoxitin (inhibition zone ≤ 18 mm) were preliminarily considered positive for AmpC β-lactamase. These potential AmpC beta-lactamase-producing isolates were subjected to a confirmatory phenotypic test using the D96C AmpC detection test based on the manufacturer’s instructions. The D96C AmpC test uses cefpodoxime to screen for chromosomal and plasmid-encoded AmpC [30]. In performing the test, a sterile needle was used to place three discs, A (cefpodoxime and Amp C inducer), B (cefpodoxime, AmpC inducer and ESBL inhibitor) and C (cefpodoxime AmpC inducer, ESBL inhibitor and AmpC inhibitor) at equitable distances on an inoculated bacterial lawn. The plates were incubated aerobically at 37 °C for 24 hours. An isolate was considered AmpC positive if the zone of inhibition of disc C exceeded that of both discs A and B by ≥ 5 mm, while zones differing by ≤ 3 mm were AmpC negative isolates. In a case where the zones of inhibition of both disc B and C exceeded that of disc A by ≥ 5 mm and those of disc B and C had a difference of ≤ 4 mm, the isolate was reported to be negative for AmpC-production but considered to be exhibiting a different resistance mechanism [29]. Molecular detection of AmpC beta-lactamases was achieved through PCR amplification of plasmid-mediated *ampC* genes encoding the phenotypes MOX, CIT, ACC, EBC, FOX and DHA (Table 2). Each PCR cycling condition comprised an initial denaturation at 95 °C for 2 minutes, followed by 30 cycles of DNA denaturation at 94 °C for 45 seconds, primer annealing at 55.9 °C for 45 seconds, primer extension at 72 °C for 1 minute and a final extension at 72 °C for 5 minutes. All PCR products were resolved by electrophoresis.

Phenotypic identification of ESBL-producing isolates was done using the D64C ceftazidime ESBL identification set (Mast Diagnostics, UK). The test was performed and results interpreted according to the manufacturer’s instructions. Similar to the D63C test, discs containing ceftazidime (30 μg) and ceftazidime/clavulanic acid (30/10 μg) were placed equitably apart on MHA inoculated with the standardised bacterial suspension. Following overnight incubation at 37 °C, the diameter of the zones of inhibition around the discs was recorded. An increase in the inhibition zone diameter of ≥ 5 mm in the presence of clavulanic acid indicated an ESBL-positive strain. ESBL producing isolates were screened for the presence of the *bla*_TEM_ gene by PCR assay using specific primers (Table 2).

### 2.5 Molecular typing of AmpC beta-lactamases producing *Escherichia coli* using ERIC-PCR

Fifteen (15) PCR confirmed AmpC beta-lactamase-producing *E*. *coli* isolates were subjected to ERIC-PCR to generate genetic fingerprints. The primer ERIC 2 (5’-AAGTAAGTGACTGGGGTGAGCG-3’) was used based on a previous report [57]. Cycling conditions included an initial denaturation at 94 °C for 2 minutes followed by 30 cycles of denaturation at 94 °C for 30 seconds, annealing at 50 °C for 1 minute and elongation at 65 °C for 8 minutes and a final extension at 65 °C for 8 minutes. The ERIC fingerprints were obtained by resolving PCR products on a 2% (w/v) agarose gel stained with 0.0001 μg/ml ethidium bromide (Bio-Rad Laboratories, Canada). Electrophoresis was performed at 60 V for 120 minutes. The gels were visualized under UV and imaged using a ChemiDoc^™^ MP Imaging system (Bio-Rad, Hercules, USA). Fingerprinting patterns were analysed using the BioNumerics software version 7.6 (Applied Maths, Sint-Martens-Latem, Belgium). *Salmonella* Braenderup H9812 was used as a control and for standardization of the gels. Band similarity was calculated by applying the dice coefficient method with an optimization of 0.5% and a band matching tolerance of 1%. Cluster analysis was performed using the unweighted pair group methods arithmetic average algorithm to construct a dendrogram. The dendrogram was further analysed for associations of isolates in the various clusters originating from the different sources.

### 2.6 Statistical analysis

Statistical analysis was performed using the Statistical Package for Social Sciences (SPSS) v. 27. The Fisher Exact test was used to determine relationships between variables and statistical significance was set at *p* = 0.05.

## 3. Results

### 3.1 Occurrence of *Enterobacteriaceae* in various samples

**Fig 1:**
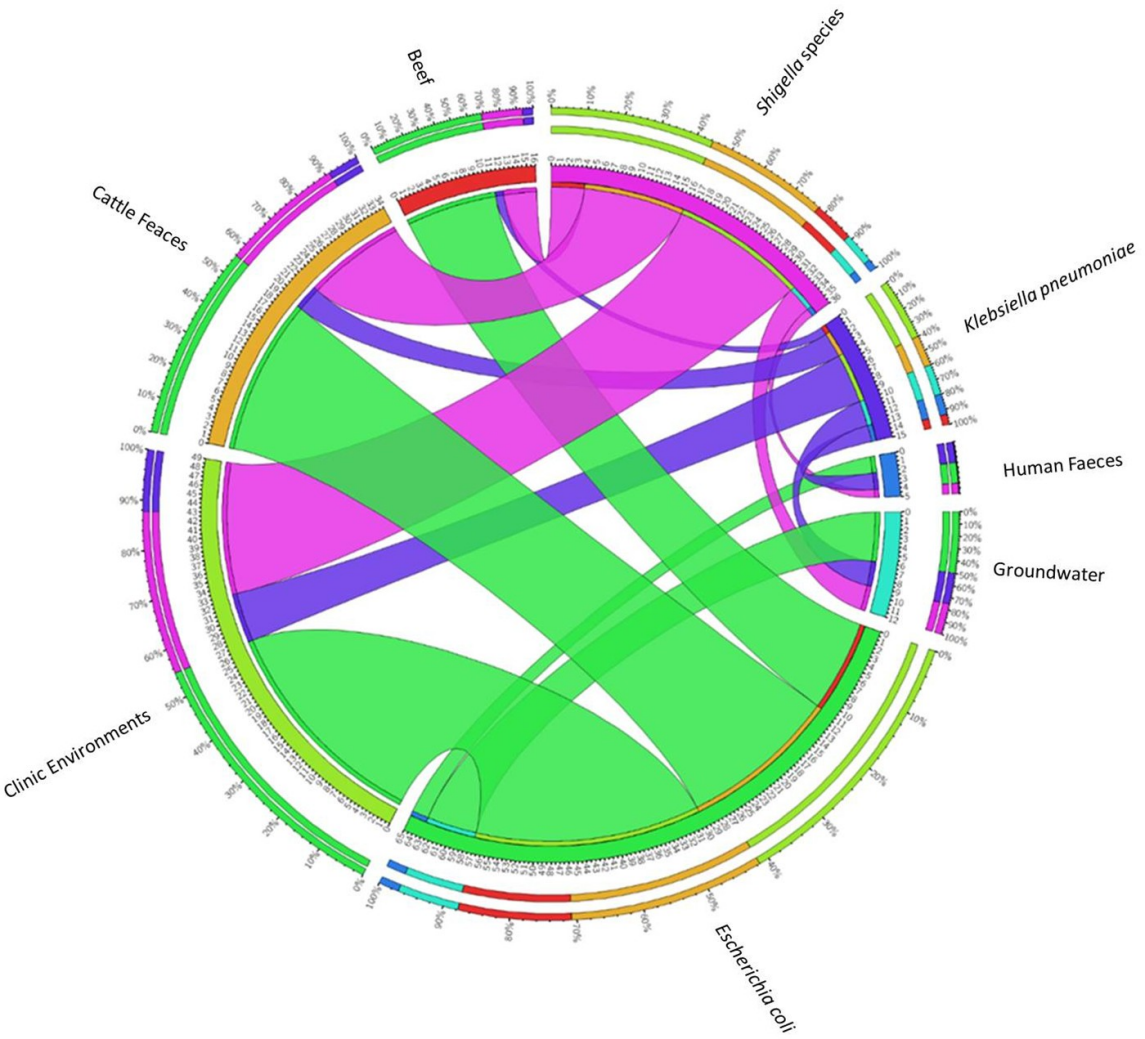
Summarized results of PCR-identified isolates from various environments.

One hundred and fifty (150) out of the 177 samples screened were positive for *Enterobacteriaceae* and included all groundwater (*n* = 40) and cattle faecal (*n* = 54) samples, 41 (64.1%) clinical swabs, 11 (91.7%) beef and 4 (57.1%) human faecal samples. PCR identification of 203 presumptive *Enterobacteriaceae* isolates resulted to a higher detection frequency for *E*. *coli* (32.0% *n* = 65), followed by *Shigella* species (26.1%, *n* = 36) and *Klebsiella pneumoniae* 15 (17.7%). The rest (42.9%, *n* = 87) were considered as other members of *Enterobacteriaceae* not considered for further analysis in the study. There was a significant difference (*p* < 0.001) in the distribution of *Enterobacteriaceae* species with respect to sample source. Fig 1 shows the distribution of various *Enterobacteriaceae* strains. Representative gels (S1-S3 Figs in S1 File) are documented in the supplementary material.

### 3.2 Antimicrobial resistance profile of isolates

Generally, resistance to the quinolone antibiotic was the most frequent, followed by resistance to the Cephems while high susceptibilities were recorded to Amikacin (Aminoglycoside) and Meropenem (Carbapenem). These patterns were observed in *E*. *coli* isolates whereby non-susceptibility was highest to Ciprofloxacin (55.4%, 36/65), followed by the cephems [Cefotaxime (43.1%, 28/65), Cefuroxime and Ceftazidime (33.8%, 22/65)]. Apart from a 29.2% (19/65) resistance recorded to Aztreonam, *E*. *coli* isolates revealed < 25% resistance to the rest of the antibiotics tested with a remarkable sensitivity (100%) to Amikacin (Table 3). Similar to the resistance observed in *E*. *coli* isolates, *Shigella* spp. were often resistant to Ciprofloxacin (44.4%, 16/36) and their resistance to Cefuroxime (36.1, 13/36), Cefepime (30.6, 11/36), Cefuroxime and Aztreonam (27.8, 10/36) were higher compared to the rest (susceptibilities of ≥ 75%) of the antibiotics tested. Ciprofloxacin was also the least effective antibiotic (53.3% resistance) against *K*. *pneumoniae*. *Klebsiella pneumoniae* isolates also exhibited a 40% resistance to Aztreonam and Ceftazidime, 33.3% to Cefotaxime, Cefepime and Cefuroxime and 26.7% to Trimethoprim-Sulfamethoxazole. Only 3 (20%) isolates in this group were resistant to Ceftriaxone and Gentamicin, 2 (13.3%) to Amikacin and Meropenem, and 1 (6.7%) to Piperacillin/Tazobactam.

**Table 3:** Number and proportion of antibiotic-resistant *Enterobacteriaceae* strains.

Bacteria species	Origin	CPM	CXM	CAZ	CRO	CTX	PTZ	MEM	GM	AK	TS	CIP	ATM
*E*. *coli* (N = 65)	CE (*n* = 27)	-	6	1	5	8	1	1	1	-	1	6	5
	CF (*n* = 19)	6	8	10	5	7	3	3	3	-	4	16	7
	BP (*n* = 11)	3	4	6	1	6	1	-	-	-	5	9	1
	BH (*n* = 6)	5	3	4	2	5	-	-	3	-	-	4	4
	HF (*n* = 2)	2	1	1	2	2	1	-	1	-	2	1	2
	Total (%)	**16 (24.6)**	**22 (33.8)**	**22 (33.8)**	**15 (23.1)**	**28 (43.1)**	**6 (9.2)**	**4 (6.2)**	**8 (12.3)**	**0 (0)**	**12 (18.5)**	**36 (55.4)**	**19 (29.2)**
*Shigella* spp. (N = 36)	CE (*n* = 16)	-	3	-	1	2	-	1	-	1	2	2	2
	CF (*n* = 12)	7	7	7	5	4	5	-	4	1	2	9	5
	BP (*n* = 4)	3	1	-	2	4	-	-	-	-	3	3	1
	BH (*n* = 3)	-	1	1	1	-	-	-	1	-	1	1	1
	HF (*n* = 1)	1	1	1	1	-	-	-	1	-	1	1	1
	Total (%)	**11 (30.6)**	**13 (36.1)**	**9 (25)**	**10 (27.8)**	**10 (27.8)**	**5 (13.9)**	**1 (2.8)**	**6 (16.7)**	**2 (5.6)**	**9 (25)**	**16 (44.4)**	**10 (27.8)**
*K*. *pneumoniae* (N = 15)	CE (*n* = 3)	-	-	-	-	-	-	-	-	-	2	1	1
	CF (*n* = 1)	-	2	2	1	-	1	2	-	2	-	3	1
	BP (*n* = 6)	1	-	1	-	1	-	-	1	-	-	1	1
	BH (*n* = 3)	2	2	2	2	2	-	-	1	-	1	2	2
	HF (*n* = 2)	2	1	1	-	2	-	-	1	-	1	1	1
	Total (%)	**5 (33.3)**	**5 (33.3)**	6 (**40)**	3 (**20)**	5 (33.3)	1(**6.7)**	2(**13.3)**	3 (**20)**	2(**13.3)**	4(**26.7)**	8(**53.3)**	6(**40)**

*Resistant (resistant + intermediate resistant). CTX = Cefotaxime (30 μg), CPM = Cefepime (30 μg), CXM = Cefuroxime (30 μg), CAZ = Ceftazidime (30 μg), CRO = Ceftriaxone (30 μg), PTZ = Piperacillin + Tazobactam (110 μg), MEM = Meropenem (10 μg), GM = Gentamicin (10 μg), AK = Amikacin (30 μg), TS = Trimethoprim-Sulfamethoxazole (25 μg), CIP = Ciprofloxacin (5 μg), ATM = Aztreonam (30 μg), CE = Clinical environment, CF = Cattle faeces, BP = Beef product, BH = Borehole (Groundwater), HF = Human faeces,

Generally, the aforementioned differences observed for most of the antibiotics tested were insignificant (*p* > 0.05), except for the resistance of the various bacterial species/groups obtained against Amikacin (*p* = 0.024). Table 3 depicts detailed results of the antimicrobial-resistant profiles of the isolates.

### 3.3 Prevalence of AmpC beta-lactamases/ ESBL among *Enterobacteriaceae* isolates

Cefoxitin disc revealed about a third of the *Enterobacteriaceae* isolates (38/116, 32.8%) as cefoxitin-resistant, considered as potential AmpC β-lactamase producers. Nineteen (50%) of these (16 *E*. *coli* and 3 *Shigella* spp.) were confirmed positive using the D69C AmpC detection test comprising 16.4% (19/116) of the overall *Enterobacteriaceae* population. A large proportion of the AmpC beta-lactamase producers harbored the *bla*_*ACC*_ gene (n = 14; 73.7%), while the *bla*_CIT_, *bla*_FOX_, and *bla*_DHA_ were detected in 5 (26%), 3 (16%), and 2 (11%) isolates respectively (Table 4). None of the isolates harboured the *bla*_ACT_ and *bla*_FOX_ genes. Only one (5.3%) isolate harboured both *bla*_ACC_ and *bla*_DHA_ genes. S4-S6 Figs in S1 File are representative agarose gels (supplementary material).

**Table 4:** Distribution of AmpC beta-lactamase producers and encoding genes among *Enterobacteriaceae* isolates.

Species	Cefoxitin disc	AmpC D96C	AmpC beta-lactamase genes detected					
			*bla*_ACC_	*bla*_ACT_	*bla*_CIT_	*bla*_DHA_	*bla*_FOX_	*bla*_MOX_
*E*. *coli* (*n* = 65)	24 (36.9)	16 (24.6%)	14	0	5	2	3	0
*Shigella* spp. (*n* = 36)	11 (30.6%)	3 (8.3%)	0	0	0	0	0	0
*K*. *pneumoniae* (*n* = 15)	3 (20%)	0 (0)	-	-	-	-	-	-
Total (N = 116)	38 (44.8)	19 (16.4%)	14 (73.7)	0 (0)	5 (26.3)	2 (10.5)	3 (15.8)	0 (0)

**Fig 2:**
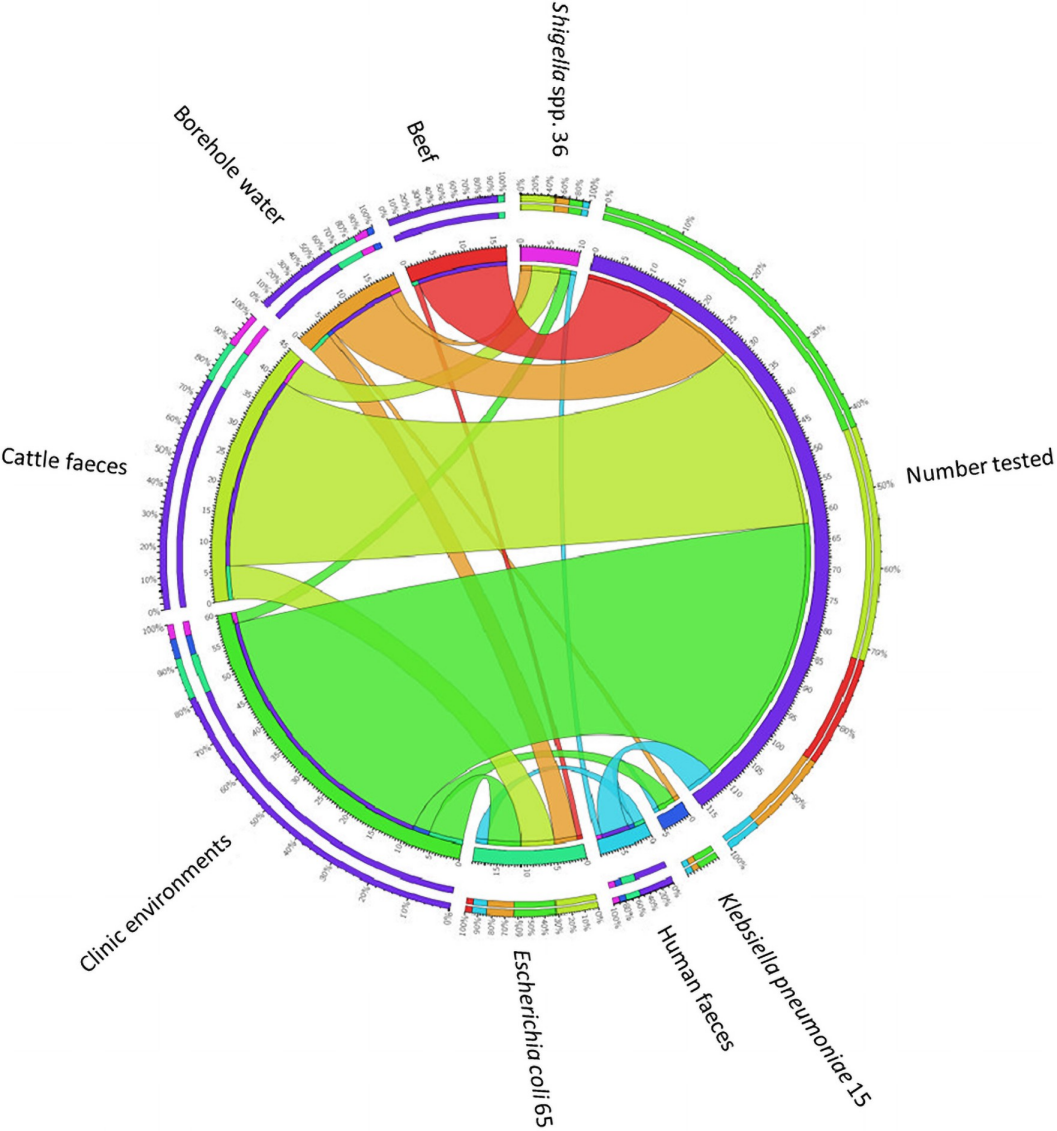
Proportion of ESBL-producing *Enterobacteriaceae*.

Out of the 116 *Enterobacteriaceae* isolates screened for ESBL phenotypes, 34 (29.31%) tested positive which comprised 19 *E*. *coli*, 10 *Shigella* spp. and 5 *K*. *pneumoniae* strains (Table 5, Fig 2). Only a small percentage (*n* = 2; 1.72%) exhibited both AmpC and ESBL phenotypic traits. A large proportion (*n* = 27; 79.4%) of the phenotypically positive ESBL isolates consisting of 15, 8 and 4 isolates of *E*. *coli*, *Shigella* spp. and *K*. *pneumoniae* respectively, harboured the *bla*_TEM_ gene (S7 Fig in S1 File).

**Table 5:** Prevalence of ESBL-producing *Enterobacteriaceae*.

Sample origin	Number tested	*E*. *coli (n = 65)*	*Shigella* spp (*n* = 36)	*K*. *pneumoniae* (*n* = 15)
Clinical environments	49	6	2	3
Cattle feces	34	6	5	0
Borehole water	12	4	2	1
Human feces	5	2	1	1
Beef	16	1	0	0
**TOTAL**	116	19 (29.2%)	10 (27.8%)	5 (33.3%)

In summarily, most of the AmpC producers were multidrug-resistant and a *Shigella* species from cattle faeces, Y10 showed resistance or intermediate resistance to all antibiotics initially tested and doubles as a co-producer of ESBL. All AmpC isolates were susceptible (17/19) or showed intermediate susceptibility (2/19) to the cephem antibiotics. A detailed summary of the characteristics of all AmpC beta-lactamase isolates detected in the study is shown in Table 6.

**Table 6:** Summary characteristics of AmpC beta-lactamase positive *Enterobacteriaceae*.

S/N	Isolate code	Bacterial strain	Source	Antibiotic resistance pattern	AmpC genes present	ESBL phenotype
1	Y6	*E*. *coli*	Cattle faeces	CTX^I^, FOX^I^, CIP^I^, ATM^I^, MEM^I^, CRO^I^, PTZ^I^	*bla*_ACC_	Negative
2	Y22	*E*. *coli*	Cattle faeces	CXM^R^, CAZ^R^, CTX^I^, CIP^I^,	*bla*_ACC_, *bla*_CIT_	Negative
3	Y11	*E*. *coli*	Cattle faeces	CTX^R^, CPM^R^, TS^R^, CIP^I^, CAZ^I^, ATM^I^	*bla*_ACC_	Negative
4	Y27	*E*. *coli*	Cattle faeces	CTX^R^, CPM^R^, TS^R^, ATM^R^ CIP^I^	*bla*_ACC_	Positive +
5	BCC	*E*. *coli*	Hospital environment	TS^I^	-	Negative
6	MCB2P	*E*. *coli*	Hospital environment	-	*bla*_CIT_	Negative
7	MPCW	*E*. *coli*	Hospital environment	FOX^R^	*bla*_ACC_	Negative
8	BCB1	*E*. *coli*	Hospital environment	CTX^R^, FOX^R^, CXM^R^, CRO^R^ MEM^I^,	*bla*_ACC_, *bla*_CIT_	Negative
9	MPCP	*E*. *coli*	Hospital environment	-	*bla*_DHA_	Negative
10	LPVAW	*E*. *coli*	Hospital environment	CXM^R^, TS^I^,	*bla*_ACC_	Negative
11	ZM2-B	*E*. *coli*	Beef product	CTX^R^, FOX^R^, CIP^R^, CXM^R^, ATM^I^, TS^I^	*bla*_ACC_	Negative
12	ZM1-B	*E*. *coli*	Beef product	FOX^R^, CXM^R^, CTX^I^, CIP^I^ CPM^I^, CAZ^I^	*bla*_ACC_, *bla*_CIT_	Negative
13	LM1-A	*E*. *coli*	Beef product	CTX^I^	*bla*_ACC_ *bla*_CIT_	Negative
14	LB1-A	*E*. *coli*	Beef product	-	*bla*_ACC_	Negative
15	ZB1-A	*E*. *coli*	Beef product	CTX^R^, CRO^R^, TS^R^, CIP^I^, CAZ^I^, PTZ^I^	*bla*_ACC_	Negative
16	LM1-C	*E*. *coli*	Beef product	TS^I^, FOZ^I^, CIP^I^, CAZ^I^,	*bla*_ACC_, *bla*_DHA_	Negative
17	Y10	*Shigella* spp.	Cattle faeces	CTX^R^, GM^R^, FOX^R^, CIP^I^, CXM^R^, CAZ^R^, ATM^R^, CRO^R^, PTZ^I^, CPM^I^, ATM^I^		Positive
18	LPCB	*Shigella* spp.	Hospital environment	CTX^R^, FOX^R^, CXM^R^	-	Negative
19	KW1-B	*Shigella* spp.	Beef product	CTX^R^, CPM^I^, TS^R^, FOX^R^, CIP^I^, CXM^R^, ATM^I^, CRO^R^		Negative

* R = resistant, I = intermediate resistance

### 3.4 Genetic relatedness of AmpC positive *Enterobacteriaceae*

**Fig 3:**
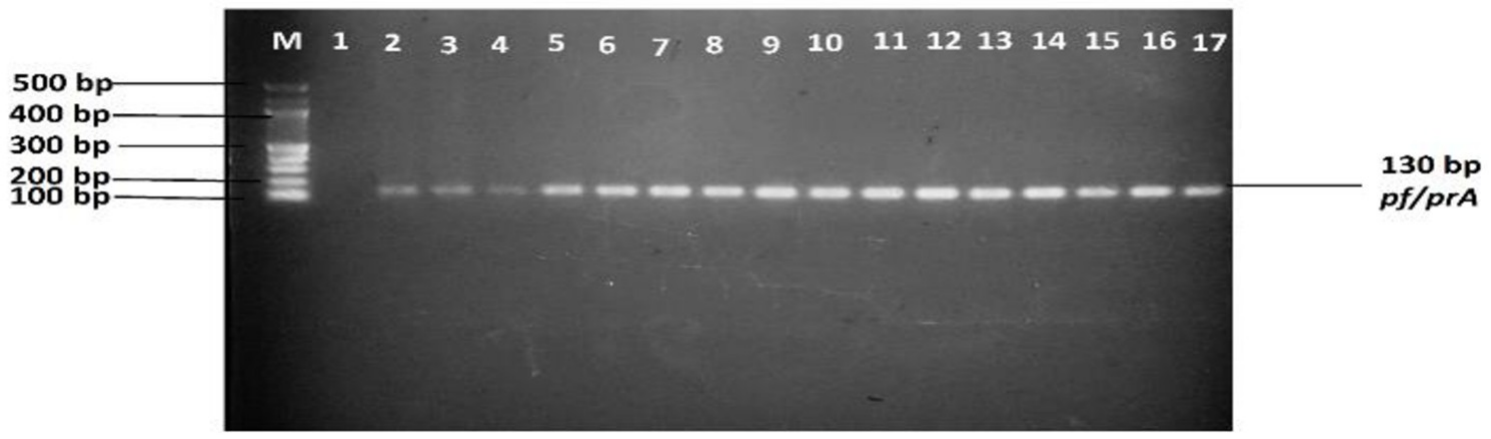
Agarose gel showing ERIC-PCR fingerprints of 15 AmpC- beta-lactamase *E*. *coli* isolates. M = 1Kb DNA molecular weight marker; Lane 1 = BCC; Lane 2 = BCB1; Lane 3 = LPVAW; Lane 4 = 6; Lane 5 = ZM2-B; Lane 6 = ZM1-B; Lane 7 = 22; Lane 8 = 11; Lane 9 = LM1-A; Lane 10 = 27; Lane 11 = ZB1-A; Lane 12 = LM1-C; Lane 13 = MCB2P; Lane 14 = MPCW; Lane 15 = MPCP; Lane 16 = LB1-A.

**Fig 4:**
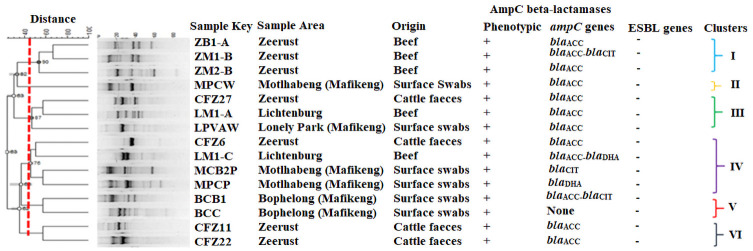
Dendrogram generated from ERIC-PCR cluster analysis of AmpC- beta-lactamase-producing *Escherichia coli* isolates.

The Fifteen AmpC beta-lactamase-producing *E*. *coli* strains typed by ERIC-PCR were of beef, clinical environment and cattle faecal origin. Generally, ERIC fingerprints ranged from 3 to 9 fragments per isolate and the relative sizes were between 250 bp to 3000 bp (Fig 3). ERIC fingerprinting profiles (Fig 3) placed the 15 isolates into 6 major clusters indicating genetic diversity with the largest cluster (cluster IV) having four isolates (one each from beef and cattle faeces samples and two from hospital swabs). On the contrary, the smallest cluster (cluster II) possessed only one isolate from a clinic environment in Motlabeng (Fig 4). However, this isolate shared some similarity with those in cluster I that mainly comprised isolates from beef samples collected from Zeerust (Fig 4). Cluster analysis revealed a 45% similarity cut off value regardless of the sampling area or source of the isolate. Fig 4 shows the dendrogram obtained from ERIC-PCR fragments.

## 4. Discussion

*Enterobacteriaceae* pathogens are implicated in intra-abdominal, urinary tract, bloodstream, and various nosocomial infections and may resist antibiotic treatment due to the production of beta-lactamases that hydrolyse beta-lactam antibiotics [19]. The presence of these pathogens in borehole water intended for human use and consumption suggests potential health risks to consumers. Boreholes are usually proved to supply pure water when first drilled with little need for treatment. However, over time, boreholes become vulnerable to pollution with increased microbial contamination. Unfortunately, especially in poor communities, there is a continued water usage without tank cleaning and treatment [58]. Moreover, boreholes are compromised by solid waste dumping sites and general littering by animal droppings from farms [59]. Even in cases where residents are aware of the possible unsafe nature of the water, they are often left with no alternatives as these are usually poverty-stricken communities without access to water purifiers [58].

Meat safety is increasingly becoming an issue of severe concern. Cattle faeces harbouring *Enterobacteriaceae* such as *E*. *coli* is not surprising. However, cross-contamination in slaughterhouses and contamination during processing and distribution due to poor handling practices may occur resulting in the detection of the strains in beef products [60]. The higher isolation frequency of *E*. *coli* compared to other *Enterobacteriaceae* is similar to a report by Gwida *et al*. [46] in which a large proportion (54%) of the isolates from raw beef were identified as *E*. *coli*. The consumption of *Enterobacteriaceae*-contaminated food or water may lead to illnesses including diarrhoea, urinary and respiratory tract infections, dysentery, and in some cases bacteraemia [61–63]. These bacteria species were also detected on surfaces of clinical environments for which both patients and personnel had been in contact, which serves as potential sources for the development of nosocomial infections. Nosocomial infections are most often associated with invasive medical procedures and present a major challenge to patients’ health [64, 65]. Bloodstream nosocomial infections caused by Gram-negative organisms such as *E*. *coli* and *Klebsiella* species have been previously reported [1, 64] Unlike with other environments, the microbial population in a clinical environment has to be in check, especially as patients are often immunocompromised and unable to combat infections effectively compared to their healthy counterparts. A noticeable percentage of *Shigella* species were also obtained during the study. Shigellosis, caused by *Shigella* infection, is a more severe form of gastroenteritis and regularly leads to the death of children under the age of five [62, 66]. *Shigella* species have earlier been detected in the study area from meat abattoirs [67] and river catchments [68], but not from clinical environments highlighting the immense epidemiological relevance of this data.

The emergence and increased dissemination of beta-lactam resistance, especially in *Enterobacteriaceae* is an impediment to antibiotic therapy and public health as a whole. In the present study, large proportions of the isolates originating from various sources displayed significant susceptibilities to the aminoglycoside; amikacin and the carbapenem; meropenem. A similar outcome of no resistance against imipenem and two aminoglycosides was obtained in a previous study among young children in the Limpopo Province of South Africa [69]. These findings are of great therapeutic significance particularly because carbapenems especially meropenem are often used as the first-line antibacterial agents for infections caused by AmpC beta-lactamase-producing *Enterobacteriaceae*. Despite the high susceptibility of AmpC beta-lactamase-producing *Enterobacteriaceae* to aminoglycosides, these antibiotics are not highly recommended for treating these infections because of their unnecessary increased toxicity [25].

The proportion of resistant isolates against the oxymino-cephalosporins; second (cefepime, cefuroxime) and third-generation cephalosporins (cefotaxime, ceftriaxone and ceftazidime) might have resulted mainly from the expression of plasmid-borne beta-lactamases (ESBL or AmpC) or to a lesser extend hyperproduction of chromosomal-encoded AmpC due to promoter or attenuator mutation [21]. The trend in which isolates from cattle most often displayed resistance to oxymino-cephalosporins and ciprofloxacin was particularly concerning as cattle are food-producing animals. Moreover, given that the projected increase in meat production from 200 million tons to 470 million tons globally by 2050 this may significantly increase the usage of antibiotics for both the prevention and remediation of livestock diseases [70]. This reliance will ultimately increase antimicrobial resistance among bacterial pathogens which in turn will not only affect the quality of food products produced from these animals but also greatly hamper therapeutic processes in humans.

Resistance to cefoxitin is useful in screening for AmpC production in *Enterobacteriaceae* such as *Klebsiella* spp. and *E*. *coli* especially in areas where ACC-1 and ACC-4 enzymes have never been detected [71]. The large proportion of the isolates positive for AmpC by the use of cefoxitin disc contradicts earlier findings of Polsfuss *et al*. [15], Helmy and Wasfi [72] and Wassef *et al*. [73] in which 9.9%, 18.2% and 5.8% of the isolates respectively were resistant to cefoxitin. The presence of transmissible *AmpC* gene (s) in the majority (15/19) of AmpC positive *Enterobacteriaceae* is a cause for concern, because these strains or their resistance traits may potentially be dispersed amongst other strains in the environment, a public health threat.

ESBL-producing *Enterobacteriaceae* have been officially recognized as pathogens of critical priority by the World Health Organization as they have over the years been seen as problematic to global public health [74]. As such, it was of importance to investigate their occurrence especially their co-existence/production with AmpC. A 29.3% (n = 34) ESBL production rate in the current study and co-existence of both enzymes in two of the isolates ranks below the proportion previously obtained in parts of South Africa: Richter *et al*. [75] obtained a 79.2% ESBL and 41.6% of AmpC in *Enterobacteriaceae* isolates from vegetables while Founou *et al*. [76] reported a 19.5% ESKAPE pathogens ESBL producers from clinical samples. Also, the percentage of detection of ESBL in this study was higher than the 9.4% detected from retail foods in China [77] but lower than the proportion (45.2%) detected from clinical strains from Sudan [42]. Although the CTX-M enzymes in ESBL producers are the most frequently detected globally [78], the TEM type enzymes are known to be horizontally transferrable with ease and are degraded by clavulanic acid [78, 79]. The significant number of ESBL producing isolates positive for the *bla*_*TEM*_ gene, do not agree with previous studies by Oduro-Mensah *et al*. [80] and Bajai *et al*. [81] who reported lower frequencies of detection; 41.8% and 48.7% respectively although comparable to a very high occurrence rate (95.1%) in penicillin-resistant *E*. *coli* isolated from young children in the Limpopo province of South Africa [69]. It is worth mentioning that ESBL producers are clinically significant because associated infections are related to high mortality, lengthened hospital stay, failure of therapy and general health cost. A study for monitoring antimicrobial resistance trends over 10 years, 2002–2011 revealed that ESBL-producing *Enterobacteriaceae* causing intra-abdominal infections increased from 2003 (about 115) to almost 16% in 2005 in Africa. However, there was a steady decrease in the later years reaching just below 10% by 2010. Unfortunately by 2011, intra-abdominal infections caused by these pathogens was on the rise (almost 12%), especially amongst patients in intensive care units [82]. In neonates and children, ESBL-producing *Enterobacteriaceae* have been estimated at 15% in Africa [83]. The high incidence of ESBL obtained herein is therefore very worrisome as ESBL-producing *Enterobacteriaceae* are often associated with various diseases and most infective bacteria are multi-drug resistant.

The ERIC-PCR of 15 isolates revealed low similarity of AmpC beta-lactamase-producing *Enterobacteriaceae*. Although *E*. *coli* have potentially transmissible AmpC beta-lactamase determinants, cross-contamination had unlikely occurred. Despite the genetic diversity and high heterogeneity, the great similarities in the drug resistance genes (identified by the presence of the *bla*_*ACC*_ gene) as shown in clusters I and IV indicate the potential to pose similar public health and/or therapeutic challenges in humans. The data generated through ERIC-PCR proved that there is a need to reduce or eradicate contamination levels in beef products by implementing strict hygiene practices during the handling of beef carcasses.

## 5. Conclusion

The increased public health threat posed by multidrug-resistant ESBL and AmpC beta-lactamase-producing strains especially among pathogenic *Enterobacteriaceae* is a cause for concern. The potential of chromosomally encoded *ampC* genes to be induced and upregulated in most *Enterobacteriaceae* when exposed to β-lactam antibiotics or other stimuli thus conferring resistance to broad-spectrum Cephalosporins as well as the potential for the rapid dissemination of plasmid-mediated *ampC* genes in other *Enterobacteriaceae* strains amplify the need for constant surveillance. In summary, the study revealed the occurrence of AmpC positive *E*. *coli*, *Shigella* species and *K*. *pneumoniae* in borehole water, beef, human and cattle faeces as well as clinical environments with high frequencies of occurrence in the latter two. Although AmpC beta-lactamases were confirmed less prevalently than ESBL, their occurrence is a cause for concern because the expression *ampC* genes in the identified *Enterobacteriaceae* strains are often carried on transmissible plasmids and may be a source of transmission to other environments and humans, as well as a source of antibiotic resistance in HA- infections, a severe health threat. Thus, strategies to ensure food safety and proper hygienic practices especially with regards to those meant for human consumption are essential. There might be a need to reassess and improve hygienic practices in cattle farms and abattoirs. Moreover, vigorous cleaning methods could be adapted as these pathogens could be thriving within the currently used methods. Continuous surveillance studies are also required for a better understanding of the clinical implications of AmpC infections, which will, in turn, aid in hospital infection control and administration of appropriate antibiotics.

## 6. Limitation of the study

In this study, Multilocus Sequence Typing (MLST) was not included as a tool to determine the genetic relatedness of isolates from different sources and this is a limitation.

## Supporting information

S1 File (PDF)
