# Supplementary material for: Evidence of potentially unrelated AmpC beta-lactamase producing Enterobacteriaceae from cattle, cattle products and hospital environments commonly harboring the blaACC resistance determinant
Source: PLoS One. 2021 Jul 29;16(7):e0253647. doi: 10.1371/journal.pone.0253647 (PMC8321102; doi:10.1371/journal.pone.0253647)
Supplement: S1 File — (PDF) [file pone.0253647.s001.pdf]

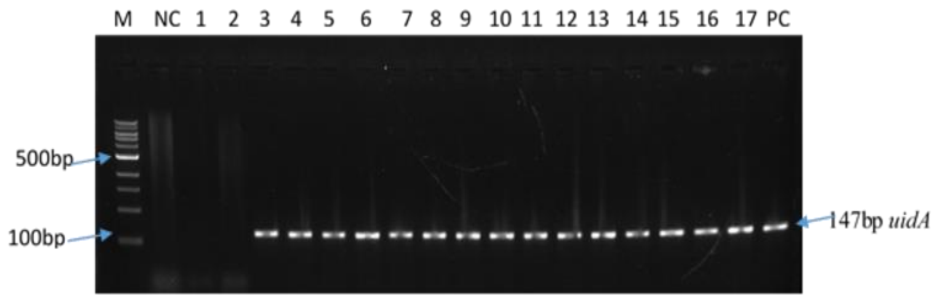

S1 Fig. **Representative agarose gel image illustrating PCR amplicons of *E. coli uidA* gene.** Lane M = 100bp molecular weight marker, Lane NC = negative control. Lanes 1 and 2 = negative isolates. Lanes 3-17 = positive isolates. Lane PC = positive control.

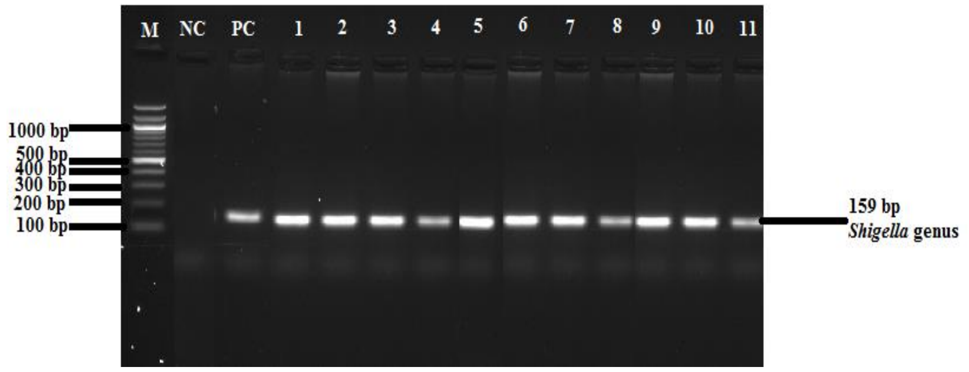

S2 Fig: **Representative agarose gel showing PCR amplicons for Shigella spp.-specific gf/r amplified gene fragment.** Lane M = 100 bp DNA molecular weight marker; Lane NC = negative control, Lane PC = positive control, Lanes 1-11 = positive gf/gr gene fragments amplified from isolates.

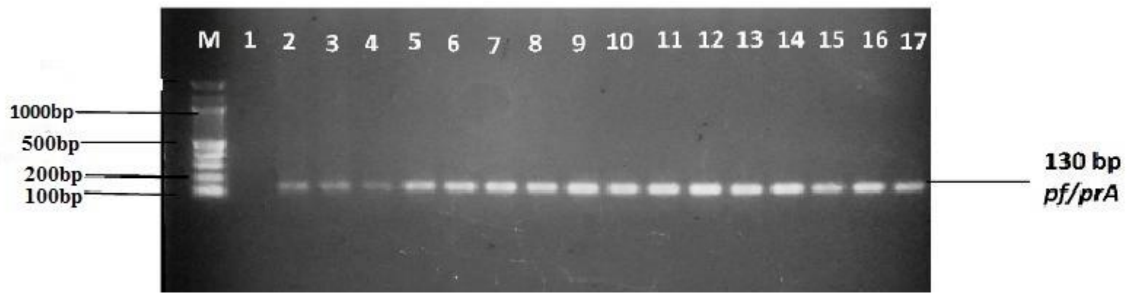

S3 Fig: **Agarose gel image illustrating PCR amplicons for the *K. pneumoniae pf/prA* positive gene fragments**. Lane M=100 bp molecular weight marker, Lane 1 = negative control isolate; Lane 2 = positive control, Lanes 3-17 = *K. pneumoniae* positive isolates.

.

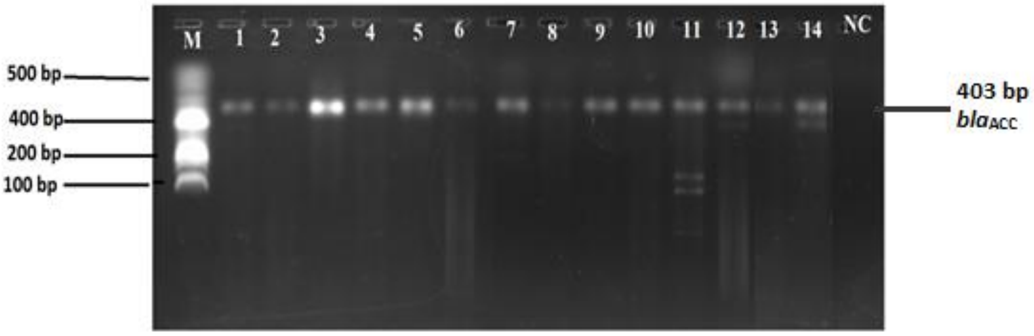

S4 Fig. An agarose gel image illustrating *bla*ACC gene fragments amplified from the isolates. Lane M = 100 bp molecular weight marker, Lanes 1-14 = positive *bla*ACC gene fragments, Lane NC = negative control.

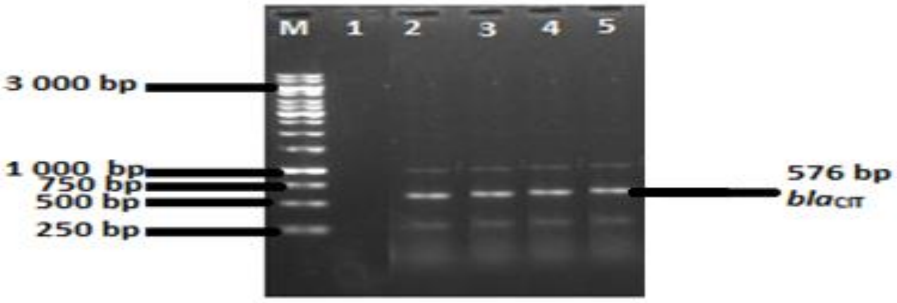

S5 Fig. Representative agarose gel image illustrating *bla*CIT gene fragments amplified from the isolates. Lane M = 1Kb molecular weight marker, Lane 1 = negative control and lanes 2-5 = positive *bla*CIT gene fragments.

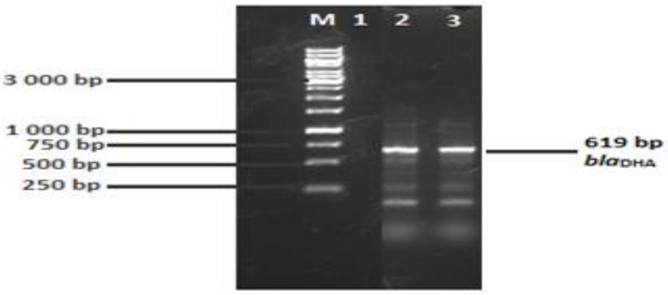

S6 Fig. Representative gel image illustrating amplified *bla*DHA gene fragments. Lane M = 1Kb Molecular weight marker, Lane 1 = positive control and Lanes 2 and 3 = positive *bla*DHA gene fragments.

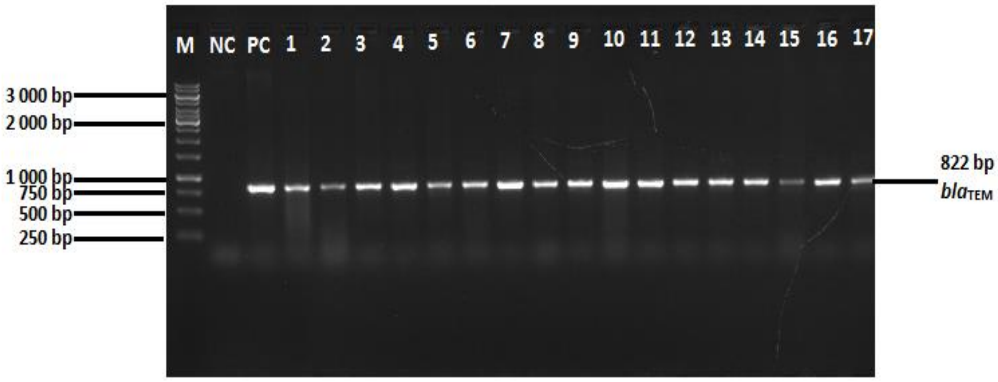

Fig S7. Agarose gel image illustrating amplified *bla*$_{\text{TEM}}$ gene fragments. Lane M = 1 Kb molecular weight marker, Lane PC = *bla*$_{\text{TEM}}$ gene fragment amplified from *E. coli* (ATCC 35218) positive control, Lane NC = negative control strain and Lanes 1-17 = *bla*$_{\text{TEM}}$ gene fragments amplified from isolates obtained in the study.
